# Obesity prevalence, physical activity, and dietary practices among adults in Saudi Arabia

**DOI:** 10.3389/fpubh.2023.1124051

**Published:** 2023-03-28

**Authors:** Salhah Alsulami, Mukhtiar Baig, Tauseef Ahmad, Nouf Althagafi, Eman Hazzazi, Razan Alsayed, Majd Alghamdi, Thikra Almohammadi

**Affiliations:** ^1^Department of Internal Medicine, Faculty of Medicine in Rabigh, King Abdulaziz University, Jeddah, Saudi Arabia; ^2^Department of Clinical Biochemistry, Faculty of Medicine in Rabigh, King Abdulaziz University, Jeddah, Saudi Arabia; ^3^Department of Epidemiology and Health Statistics, School of Public Health, Southeast University, Nanjing, China; ^4^Faculty of Medicine in Rabigh, King Abdulaziz University, Jeddah, Saudi Arabia

**Keywords:** obesity, overweight and obesity, physical activity, dietary habits, lifestyle factors

## Abstract

**Introduction:**

The current study evaluated obesity prevalence, physical activity, and dietary practices among Saudi adults in the Makkah region of the Kingdom of Saudi Arabia (KSA). The current survey was accomplished between November 2021 and March 2022.

**Method:**

A validated questionnaire, Arab Teens Lifestyle Study (ATLS), was used to evaluate all participants' physical activities, sedentary behaviors, and nutritional habits in addition to demographic data.

**Result:**

A total of 2,115 people [1,238 (58.5%) women and 877 (41.5%) men] participated in this survey. Being overweight was prevalent in 32.8% of the population (41% of men and 28.9% of women), obesity was prevalent in 23% of the population (males 23.1% and females 24.2%). Obese people consumed more soft drinks, and overweight people did not consume enough vegetables (fresh/cooked). Obese people consumed fast food (e.g., burgers, sausage, pizza, or Arabic shawarma) over three times each week. The mean (SD) number of days of practice walking was 2.51 (2.05) vs. 1.3 (1.87) (*p* < 0.001) for lean and obese individuals, respectively. In addition, individuals with normal BMI had more days of jogging, moderate and high-intensity exercise, dancing, and strength training than those with obesity. The odds of being obese increased with age (OR: 1.07; *p* < 0.001), in males (OR: 2.16; *p* < 0.001), in participants earning <5,000 SR/month (1.3 thousand $) and 10–15 thousand SR/month (1.34–2.66 thousand $) (OR: 2.36; *P* = 0.01). Obesity was inversely associated with moderate-intensity exercise (OR: 0.802; *p* = 0.009), and regular walking (OR: 0.685; CI: 0.624–0.752; *p* < 0.001).

**Discussion:**

Overweight and obesity were prevalent in 32.8% and 23% of the population, respectively. Sociodemographic factors associated with obesity. Focused intervention strategies are needed to overcome the obesity issue.

## Introduction

Scientific discoveries have made every facility available at our fingertips, causing us to adopt sedentary lifestyles and physical inactivity. The advancement of technology, on the one hand, provided relief, conveniences, and pleasures and improved quality of life; on the other hand, being overweight and obese is one of its banes. Obesity has nearly tripled globally since 1975 (1). This is almost a 50-year period when scientific progress is at its peak and enveloped our physical activities. In the recent era, it has become a global epidemic issue.

Globally, over 1.9 billion adult individuals (39%) were overweight, and over 650 million people (13%) were obese (10.8% of men and 14.9% of women) in 2016 (2). The World Health Organization (WHO) reported that the overweight and obesity prevalence in KSA is 68.2% (women 69.2% and men 67.5%) and 33.7% (women 39.5% and men 29.5%), respectively (3). A recent large survey collected data from all regions of KSA and revealed a 24.7% obesity prevalence (4).

Weight status is often thought to result from bad eating habits, a sedentary lifestyle, or a mix of both, given its rising frequency among youngsters (5). Obesity is a complex multidimensional issue influenced by numerous modifiable and non-modifiable elements, including hereditary, demographic, and lifestyle factors (6). It has grown into a huge public health issue because of the increased probability of acquiring type 2 diabetes mellitus, hypertension, cardiovascular disease, obstructive sleep apnea, gall stones, hyperlipidemia, fatty liver disease, osteoarthritis, and psychosocial consequences (4, 7, 8). The current global expenses of obesity are projected to be more than US $2 trillion each year, combining direct healthcare costs and lost economic output, which amounts to 2.8% of global GDP (9).

There is growing evidence linking obesity with vicissitudes in eating practices, primarily because of consuming low quantities of vegetables, fruits, and grains; a constant rise in processed foods intake; increased consumption of sugary beverages and other sugary items, and dining away from home. These changes dramatically boost energy, resulting in a significant shift in metabolic health (10, 11). Most Asian people have a positive energy balance because of lower physical activity at their jobs due to automation, improved transportation, and spending most of their time watching television (12, 13). Many experts attributed obesity as a transfer from indigenous eating habits to western diet habits consisting of ultra-processed foods; therefore, it is considered a legacy of society's modernization (14, 15). These changes have permeated the societies of the Middle East region during the last few decades.

Obesity is a mounting public health issue in the KSA, as it is in the rest of the globe. Several studies have been published concerning the relationship between Saudi adult populations' lifestyle patterns and demographic features with overweight and obesity in various regions of the Kingdom; however, several campaigns have been launched at the governmental level in the recent past to alleviate the Kingdom's alarming rates of overweight and obesity (7, 15). The Makkah region was chosen for this study because it has a high prevalence of obesity among its inhabitants (7). Furthermore, it is one of the top five obesity and overweight regions in the KSA (4).

Because food patterns and physical activities do not remain constant. These are always changing based on age, gender, and responsibilities. Such studies aid in identifying the state of the problem and recommending some adopting steps to control and manage the problem. As a result, the current study will bring the latest updates on the topic in the existing literature, bridging the gap in research and guiding the future management of obesity.

Obesity, which has a significant impact on persons' quality of life in KSA, can be avoided by following correct eating patterns and engaging in regular exercise. Individuals' physical activities and dietary practices can assist to identify their risk for specific diseases, such as diabetes mellitus, and motivate them to adjust their lifestyle habits and care for their bodies and health. Several research investigated weight, nutritional, and physical activity concerns among KSA residents and reported mixed results.

Furthermore, the general public's apathy and lack of initiative in obesity prevention may be due to a lack of cognizance. The cornerstones of preventive and early intervention to tackle the obesity pandemic in KSA include adequate knowledge, optimism, and a proactive commitment to healthy behavior. The study hypothesized that the prevalence of obesity is high in this region and that various dietary, demographic, and lifestyle factors contribute to this high prevalence.

Considering the preceding observations, the current study sought to explore changes in obesity-related indicators among residents of the Makkah region. Therefore, the current survey's objectives were to investigate obesity prevalence, physical activity, and dietary practices among adults in the Makkah region of the KSA.

## Methods

The present analytical investigation was carried out in KSA's western region between November 2021 and March 2022 using a cross-sectional design. The ethical approval was taken from “the Biomedical Ethics Research Committee of King Abdulaziz University, Jeddah” (Reference No. 471-21). The current research was carried out in line with the Helsinki Declaration. All data were collected after taking informed consent from all the study participants. The inclusion criteria were people aged 18 years or more, who are residing in the Makkah region, and who agreed to take part in the survey. People who had any disability or suffering from any chronic disease were excluded. A total of 2,115 people were randomly chosen from various public places such as universities, parks, and shopping malls to complete a self-administered questionnaire, and no monetary or any other benefit was offered to them, and their participation was completely voluntary. The first section of the questionnaire consisted of demographic information such as age, gender, educational level, monthly income, and place of residence. Self-reported anthropometric measurements such as height, weight, and most recently measured blood pressure were included in the second section. “The BMI was computed by dividing the weight in kilograms by the square of the individual's height in meters (kg/m^2^) and divided into four groups according to the WHO criteria; (a) underweight (BMI 18.5 kg/m^2^), (b) normal weight (BMI 18.5–24.9 kg/m^2^), (c) overweight (BMI 25–29.9 kg/m^2^), and (d) obese (BMI 30 kg/m^2^ or more)” (16).

## Research instrument

ATLS questionnaire was used to obtain data about the participants' lifestyles (17). It is a 47-item survey. The first five items were related to age, height, weight, waist circumference, and education level, and the remaining were related to physical and sedentary activities and eating practices. The questions were scored using a Likert scale.

### Physical activities assessment

In total, 29 items (6–34) were related to physical activities such as exercise, swimming, light, moderate, and high-intensity sports activities, participation in traditional dancing, and housework. The original questionnaire has already been found to have good reliability “[intraclass correlation coefficient (ICC) = 0.85; 95% confidence limits (CL), 0.70–0.93] (18) and satisfactory validity (*r* = 0.37, *p* < 0.001) vs. the pedometer-measured level of physical activity” (19).

### Sedentary behaviors assessment

Three items of sedentary behavior questions (35–37) were designed to elicit essential information from adults about their average daily time devoted to sedentary activities, such as watching TV, playing video games, and benefiting from computers and the Internet. Individuals were enquired to affirm their average daily work hours, regardless of whether they worked during the week or on weekends.

### Eating habits assessment

The ALTS questionnaire assessed eating habits by 10 items (38–47). “Participants were asked frequency of breakfast, fizzy drinks (including soft drinks), vegetables, fruits, and dairy products, donuts and cakes, candy and chocolate, energy drinks, and fast foods per week. In this perspective, fast foods contained examples from both Western fast meals and Arabic fast foods, such as shawarma (grilled meat or chicken on pita bread with some salad).” These questions focused on both good and bad eating habits.

### Statistical analysis

Excel spreadsheets and SPSS version 20 were used to enter and process all data. Means and standard deviations for BMI were compiled. By using the chi-square test at the 95% level of significance, the basic relationship between sociodemographic factors and body weight status was examined. An independent t-test was employed to compare continuous variables between lean and obese groups. To evaluate obesity-related factors, multiple logistic regression analyses were performed. The model included age, gender, educational achievement, social position, numerous physical activities, and varied food habits as independent factors, and the odds ratio (OR) was computed. A level of significance of *p* < 0.05 was judged statistically significant.

## Results

Overall, 2,115 people [1,238 (58.5%) women and 877 (41.5%) men] participated in this survey. The mean age (SD) was 33.52 (13.12). Being overweight was prevalent in 32.8% of the population (41% of men and 28.9% of women), obesity was prevalent in 23% of the population (men 23.1% and women 24.2%), and being underweight was prevalent in 7.5% of the population. A total of 1,260 (59.3%) participants have a bachelor's degree and 1,829 (86.5%) live with their families (Table 1).

**Table 1:** General characteristics of the study participants (*n* = 2,115).

**Variables**	**Mean (SD)**
Age (years)	33.52 (13.124)
Mean weight (kg)	70.01 (17.844)
Mean BMI (kg/m^2^)	26.23 (6.184)
Waist circumference (cm)	76.20 (18.162)
Systolic BP (mmHg)	122.39 (13.759)
Diastolic BP (mmHg)	79.03 (9.125)
**Variables**	**Number (percentages)**
**Gender**	
Male	877 (41.5%)
Female	1,238 (58.5%)
**Nationality**	
Saudi	1,765 (83.5%)
Non-Saudi	350 (16.5%)
**Living place**	
Jeddah	1,019 (48.2%)
Rabigh	546 (25.8%)
Other cities	550 (26.0%)
**Social status**	
Married	1,066 (50.4%)
single	945 (44.7%)
Widow	30 (1.4%)
Divorce	74 (3.5%)
**Educational level**	
illiterate	34 (1.6%)
High school or below	639 (30.1%)
Bachelor	1,260 (59.3%)
Postgraduate studies	182 (8.6%)%)
**Living with**	
Family	1,829 (86.5%)
Separate	197 (9.3%)
Friends	67 (3.2%)
Others	22 (1.0%)
**Monthly income (in Saudi Riyals)**	
< 5 thousand	818 (38.7%)
5–10 thousand	498 (23.5%)
10–15 thousand	392 (10.0%)
15–20 thousand	212 (18.5%)
More than 20 thousand	195 (9.2%)
**BMI categories**	
Underweight	160 (7.5%)
Normal	712 (33.5%)
Overweight	698 (32.8%)
Obese	488 (23.0%)

There were no discernible changes in fresh fruit, dairy products, or sweet consumption between BMI categories. Overweight people did not consume enough vegetables (fresh/cooked) and ate fast food (e.g., burgers, sausage, pizza, or Arabic shawarma) on more than three occasions every week. People with normal weight consume more fresh fruit than those who are obese or overweight. Similarly, participants of normal weight ate breakfast regularly, even though the *p*-value was not significant (*p* = 0.789) (Table 2).

**Table 2:** Correlation of dietary habits with different BMI categories (normal, overweight, and obese).

**Dietary habits**	**Normal**	**Overweight**	**Obese**	***p*-value**
	***N*** **(%)**	***N*** **(%)**	***N*** **(%)**	
**Eating breakfast**				
Do not eat	69 (31.4%)	71 (32.3%)	61 (27.7%)	0.789
Eating regularly	335 (35.4%)	316 (33.4%)	221 (23.3%)	
Eating sometimes	299 (34.6%)	299 (34.6%)	202 (23.4%)	
**Drinking soft drinks**				
Do not drink	219 (35.9%)	198 (32.5%)	138 (22.6%)	0.070
1–3 times/week	373 (35.3%)	362 (34.2%)	241 (22.8%)	
More than 3 times/week	108 (30.5%)	121 (34.2%)	105 (29.7%)	
**Eating vegetables (fresh or cooked)**				
Do not eat	57 (29.1%)	78 (39.8%)	45 (23.0%)	
Eating 1–3 times/week	407 (34.0%)	401 (33.5%)	298 (24.9%)	0.399
Eating more than 3 times/week	225 (36.9%)	202 (33.1%)	136 (22.3%)	
**Eating fruits**				
Do not eat	106 (30.9%)	116 (33.8%)	88 (25.7%)	0.256
Eating 1–3 times/week	447 (35.4%)	428 (33.9%)	306 (24.2%)	
Eating more than 3 times/week	136 (35.5%)	129 (33.7%)	83 (21.7%)	
**Consuming diary product**				
Do not eat	71 (30.9%)	73 (31.7%)	65 (28.3%)	0.397
Eating 1–3 times/week	357 (34.4%)	363 (35.0%)	248 (23.9%)	
Eating more than 3 times/week	252 (35.6%)	236 (33.3%)	162 (22.9%)	
**Consuming fast food**				
Do not eat	184 (35.8%)	161 (31.3%)	117 (22.8%)	0.05
Eating 1–3 times/week	419 (34.6%)	422 (34.8%)	285 (23.5%)	
Eating more than 3 times/week	80 (31.5%)	87 (34.3%)	74 (29.1%)	
**Consuming French fries/potato chips**				
Do not eat	221 (36.5%)	198 (32.7%)	139 (23.0%)	0.312
Eating 1–3 times/week	410 (35.1%)	390 (33.4%)	283 (24.2%)	
Eating more than 3 times/week	52 (26.5%)	75 (38.3%)	53 (27.0%)	
**Eating cakes/biscuits/donuts**				
Do not eat	172 (35.8%)	164 (34.1%)	105 (21.8%)	0.845
Eating 1–3 times/week	412 (34.2%)	407 (33.8%)	295 (24.5%)	
Eating more than 3 times/week	94 (32.3%)	97 (33.3%)	77 (26.5%)	
**Sweats/chocolate**				
Do not eat	143 (34.4%)	140 (33.7%)	94 (22.6%)	0.717
Eating 1–3 times/week	412 (34.8%)	407 (34.4%)	282 (23.8%)	
Eating more than 3 times/week	123 (33.5%)	119 (32.4%)	95 (25.9%)	
**Energy drinks**				
Do not drink	523 (34.9%)	505 (33.7%)	356 (23.7%)	0.899
Drinking 1–3 times/week	125 (33.4%)	128 (34.2%)	89 (23.8%)	
Drinking more than 3 times/week	29 (33.7%)	29 (33.7%)	24 (27.9%)	

Many government employees were overweight or obese. Sociodemographic factors such as gender, nationality, social status, educational level, occupation status, and monthly income had a significant impact. Men were more overweight compared to women; on the other hand, more women were obese compared to men. Interestingly, obesity was more prevalent among housewives. Among Saudi and non-Saudi participants, obesity rates are 24.9% and 17.5% and overweight rates are 30.5% and 51.2%, respectively. Obesity is influenced by monthly income; 44.5% of people earning more than SR 20,000 are overweight. On the other hand, 39.5% of people earning < SR 5,000 per month (1.3 thousand $) are of normal weight (Table 3).

**Table 3:** Comparison of demographic factors with different BMI categories (normal, overweight, and obese).

**Factors**	**Normal**	**Overweight**	**Obese**	***p*-value**
	***N*** **(%)**	***N*** **(%)**	***N*** **(%)**	
**Gender**				
Male	255 (29.9%)	350 (41.0%)	197 (23.1%)	**0.00**
Female	457 (38.0%)	348 (28.9%)	291 (24.2%)	
**Nationality**				
Saudi	622 (36.2%)	523 (30.5%)	428 (24.9%)	**0.00**
Non-Saudi	90 (26.3%)	175 (51.2%)	60 (17.5%)	
**Social status**				
Married	253 (24.3%)	431 (41.3%)	321 (30.8%)	**0.00**
Single	431 (47.0%)	238 (26.0%)	129 (14.1%)	
Widow	8 (28.6%)	9 (32.1%)	10 (35.7%)	
Divorced	20 (28.6%)	20 (28.6%)	28 (40.0%)	
**Educational level**				
Illiterate	8 (26.7%)	13 (43.3%)	6 (20.0%)	**0.00**
Secondary and below	229 (37.2%)	192 (31.2%)	150 (24.4%)	
Bachelor student	435 (35.2%)	401 (32.5%)	293 (23.7%)	
Postgraduate	40 (22.6%)	92 (52.0%)	39 (22.0%)	
**Occupation**				
Health care worker	48 (34.5%)	55 (39.6%)	30 (21.6%)	**0.00**
Government	112 (24.7%)	179 (39.5%)	152 (33.6%)	
Private	71 (27.7%)	107 (41.8%)	59 (23.0%)	
Free work	33 (34.4%)	41 (42.7%)	21 (21.9%)	
Housewife	96 (27.7%)	117 (33.7%)	120 (34.6%)	
Unemployed	43 (38.4%)	29 (25.9%)	21 (18.8%)	
Student	309 (47.2%)	170 (26.0%)	85 (13.0%)	
**Monthly income**				
< 5 thousand	312 (39.5%)	204 (25.9%)	172 (21.8%)	**0.00**
5–9.9 thousand	168 (34.6%)	178 (36.6%)	111 (22.8%)	
10–14.9 thousand	103 (26.9%)	148 (38.6%)	118 (30.8%)	
15–20 thousand	68 (32.5%)	83 (39.7%)	49 (23.4%)	
Above 20 thousand	61 (31.9%)	85 (44.5%)	38 (19.9%)	

All values in bold are statistically significant.

The mean (SD) number of days of practice walking was 2.51 (2.058) vs. 1.30 (1.871); *p* < 0.001 for normal and obese individuals, respectively. In addition, individuals with normal BMI had more days of jogging, moderate and high-intensity exercise, dancing, and strength training than those with obesity (Table 4).

**Table 4:** Comparison of different types of physical activity in lean and obese subjects.

**Types of physical activities**	**Lean (BMI = 18.5–24.9 kg/m^2^)**	**Obese (BMI ≥30 kg/m^2^)**	***p*-value**
	**Mean (SD)**	**Mean (SD)**	
How many days per week do you exercise walking?	2.51 (2.058)	1.30 (1.871)	**0.00**
How many days a week do you regularly exercise (jogging or running or both), whether on the ground or a treadmill? (days/week)	1.24 (1.872)	0.63 (1.429)	**0.00**
How many days a week do you regularly ride a regular bike, stationary bike, or both? (days/week)	0.39 (1.156)	0.28 (0.965)	0.094
How many days a week do you swim regularly? (days/week)	0.29 (0.944)	0.23(0.945)	0.330
How many days a week do you regularly practice moderate-intensity, non-strenuous sports activities other than the activities (such as volleyball, table tennis, bowling, badminton, etc.) (days/week)	0.58 (1.306)	0.31 (0.983)	**0.00**
How many days a week do you do high-intensity and physically strenuous sports other than the activities (such as basketball, handball, football, tennis, squash, CrossFit, etc.) (days/week)	0.51 (1.373)	0.29 (0.956)	**0.002**
How many days a week do you regularly? practice self-defense sports such as (judo, karate, taekwondo)? (days/week)	0.19 (0.850)	0.17 (0.769)	0.686
How many times per week do you regularly do strength training (weight training or body building or calisthenics exercise)? (days/week)	0.70 (1.569)	0.50 (1.286)	**0.017**
How many times per week do you do traditional dancing (whether alone or with your friends)? (days/week)	0.99 (1.734)	0.71 (1.473)	**0.016**
How many times per week do you engage in household work (e.g., gardening, vacuuming, washing, car cleaning)? (days/week)	2.26 (2.575)	2.05 (2.576)	0.192
Average sleeping time (hours)	7.04 (1.853)	6.86 (1.971)	0.110

Mean days of practicing specific physical activity (SD). All values in bold are statistically significant.

The odds of being obese increased with age (OR: 1.07; *p* < 0.001), in men (OR: 2.16; *p* < 0.001), and in participants' earning < 5,000 SR/month (1.3 thousand $) (OR: 1.037; *p* = 0.001) and earning 10–15 thousand SR/month (1.34–2.66 thousand $) (OR: 2.36; *p* = 0.01). Obesity was inversely associated with moderate-intensity exercise (OR: 0.802; *p* = 0.009) and regular walking (OR: 0.685; *p* < 0.001) (Table 5).

**Table 5:** Association of demographic characteristics and other variables with different BMI.

**Characteristics**		**B**	**Obese**	
			**OR 95% CI**	***p*** **-value**
Age		0.074	1.077 (1.057–1.098)	**0.000**
Gender	Male Female	0.771	2.162 (1.540–3.036) Reference	**0.000**
Social status	Married Single Widow Divorce	0.063 0.277 0.388	1.433 (0.700–2.933) 0.758 (0.345–1.664) 0.679 (0.182–2.533) Reference	0.764 0.764 0.237 0.686
Occupation	Health care worker Government Private Free Housewife Unemployed Student	0.473 0.659 0.646 0.461 0.234 0.784	1.298 (0.637–2.648) 1.156 (0.608–2.200) 1.15 (0.631–2.103) 0.730 (0.315–1.688) 1.474 (0.778–2.792 1.106 (0.539–2.268) Reference	0.057 0.368 0.087 0.794 0.158 0.410
Place of living	Jeddah Rabigh Other cities	−0.058 −0.044	0.944 (0.665–1.341) 0.957 (0.639–1.435) Reference	0.748 0.833
Monthly income	< 5 thousand 5–9.9 thousand 10–14.9 thousand 15–20 thousand More than 20 thousand	1.073 0.432 0.861 0.277	2.925 (1.526–5.606) 1.540 (0.808–2.936) 2.366 (1.233–4.539) 1.319 (0.946–2.684)	**0.001** 0.981 **0.010** 0.444 Reference
Educational level	No grade completed Secondary or below Bachelor's Postgraduate	−1.273 −0.259 −0.210	0.280 (0.059–1.332) 0.772 (0.402–1.482) 0.810 (0.446–1.471)	0.110 0.436 Reference
Walking		−0.378	0.685 (0.624–0.752)	**0.000**
Moderate intensity exercise		−0.220	0.802 (0.680–0.946)	**0.009**
Consumption of fast food	I don't eat 1–3times/week More than 3 times/week	0.153 0.041	1.165 (0.641–2.118) 1.042 (0.639–1.699)	0.616 0.968 Reference
Drinking sugary drinks/soft drinks e.g., Coke, Pepsi	I don't drink 1–3 times/week More than 3 times/week	−0.421 −0.171	0.884 (0.53–1.455) 0.843 (0.547–1.298)	0.627 0.438 Reference

Reference category: normal BMI. All values in bold are statistically significant.

## Discussion

Obesity has generally become a huge point of concern on a global scale, and in the KSA, its mounting prevalence among children, adolescents, and adults has made it a major worry. According to the current study findings, 32.8% of the population was overweight, 23% were obese, and 7.5% were underweight. A bigger percentage of men than women were overweight, but a greater percentage of women than men were obese.

Our overweight and obesity prevalence results corroborate with Saudi, Iraqi, and Brazilian studies (4, 20, 21). However, Iraqi and Brazilian studies have reported a lower incidence of underweight individuals, 1.5% and 3.6%, respectively (20, 21), while a Saudi survey reported a much higher incidence of 21.7% (4). Several KSA studies have shown the varying prevalence of obesity and overweight (7, 22, 23). The actual prevalence of obesity may be more in KSA than in the present study findings. The possible reason could be our study participants' low mean age (33.5 years). Few studies have demonstrated that obesity and overweight increase with age (24–26). Male obesity has been linked to infertility due to a drop in sex hormone levels (27), and it induces polycystic ovarian syndrome in women by interacting with adipokine and steroid hormones (28). Obesity has a deleterious impact on female fecundity as well as fetuses and embryos *via* oxidative processes (29).

The present study results indicate the ineffectiveness of educational campaigns against obesity prevention in the region. Moreover, obesity and its consequences also have a negative impact on the healthcare budget. Therefore, the health ministry's continual campaign on social and mass media is much needed regarding this issue.

The high frequency of overweight among men (41%) is alarming. It needs immediate remedial steps because all these overweight people are at risk of becoming obese. These individuals are needed to change their dietary patterns and adopt healthy lifestyles. It is suggested that walking and other moderate-intensity exercises may help in reducing the risk of obesity (30).

Our investigations revealed that obesity is more widespread in women than men, which conforms with the results of prior investigations (20, 31, 32). Women are more obese for a variety of reasons, including their parity and biological factors such as hormonal shifts and usage of contraceptive pills (33), the less physical effort required in their employment, more household work, less spare time, and imbalance of excessive calorie intake and insufficient activities (11, 31, 34, 35). A prior study demonstrated that the female gender, old age, and reduced physical activity were all major obesity risk elements in South Asian people (36).

It is challenging to interpret why there were no significant variations in fresh fruit, dairy products, or sweet consumption across BMI categories among the current study participants. However, the overweight/obese individuals probably have some realization of their increasing weight, thus they were avoiding the consumption of too many sweets; another probability is that they underestimated their consumption of sweets.

Similar to the present study, a previous Saudi study used the same ATLS questionnaire and found no significant differences in most lifestyle-related patterns between lean and overweight/obese women (22). According to a Saudi survey, the majority of university students drank soft/energy drinks more than once a day, ate fast food daily, and spent 3–4 h per day on their screens (37).

There is emerging evidence linking dietary preferences to body weight. Few studies identified a link between bad dietary habits and being overweight or obese (38–40), but an Iraqi study found no link (21). Few studies have reported that body weight was positively linked with fast food (38) and sweetened beverages (39), and inversely linked with eating fruits, vegetables, legumes, and nuts (40). Several studies in the general population found that greater adherence to healthy eating patterns was related to a decreased incidence of obesity (44, 45).

The current study noted that obese people consumed more soft drinks and fast food such as burgers, sausages, pizza, or Arabic shawarma. There is a need to make them aware that consuming junk food is not good for their health and positively impacts weight gain. Such food also contains more saturated fats, trans-fatty acids, and decreased micronutrients that cause several health issues. As a result, consuming these items regularly may result in excess of daily calorie intake, resulting in a higher frequency of overweight and obesity and more probability of getting chronic non-communicable illnesses such as T2DM, CVD, hypertension, and others. Therefore, providing nutritional counseling and intervention concerning healthy eating is essential.

The present study results indicated that obese individuals were less active, and several sociodemographic factors were associated with obesity. Moderate-intensity exercise and walking were protective factors against obesity, and people who did not take soft drinks were less likely to be obese. Contrary to our findings, a few investigations discovered no association between demographic and lifestyle variables with overweight or obesity (7, 20). A Brazilian study observed sociodemographic characteristics (such as the mother's education level, age range, and gender) were linked to inappropriate eating situations at breakfast and/or dinner (46). Our results are compatible with several studies (20, 47). Our study found that married individuals had probability to be obese than unmarried individuals. It is because married people are older and body weight usually increases with age. Housewives had a higher prevalence of obesity, and it could be because they stay at home, and in KSA, people's socioeconomic conditions are good, and most of them have several servants and maids who perform all house chores. Because of this good socioeconomic condition, they dine outside the home more frequently and are more prone to sedentary behavior. So, this sedentary behavior tends to increase obesity among them.

Similar to our results, an Iraqi study reported that obesity and physical exercise have an inverse relationship (21). Several pieces of research have shown a relationship between physical inactivity and obesity (19, 48, 49). Our findings showed that men had more probability to be obese compared to women. This is consistent with few studies (19, 21) and contradictory with few studies that found its connection with the female gender (7, 22, 50, 51). Physical activities such as moderate exercise and regular walking contribute to the reduction of obesity. This observation could be attributed to the fact that physical activities usually burn calories, and burning more calories is beneficial for maintaining a healthy weight. Regular walking and moderate exercise improve body endurance and fitness level, as well as have a positive impact on all physiological systems. Some of the benefits of physical activity include increased mitochondrial function, better vasculature, and the production of myokines from skeletal muscle that support or enhance cardiovascular function (52). When physical activity is prolonged and muscle glycogen stores are depleted, fat metabolism may become a more important source of energy. As a result, weight loss is attainable (53). Therefore, people should take time out of their hectic lives to improve their health.

Obesity is a big issue in the KSA, and it urgently requires preventative strategies to address an increase in obesity prevalence. People must forsake sedentary behaviors and exercise regularly and eliminate extra fatty and sugar-containing foods from their regular menu to live a healthy life. There is a need to establish more walking tracks and sports centers across the city. The use and assessment of evidence-based therapies are strongly encouraged (7).

Cattafesta et al. (20) have suggested that because obesity has various causes, coping solutions must be multifaceted, intersectoral, and interdisciplinary. Implementing preventive measures, such as programs promoting a healthy diet, proper food policy, encouraging physical activity and discouraging sedentary behavior, and community awareness programs may help reduce obesity (21).

We believe that obesity can be culminated by modifying dietary patterns and providing healthier dining situations for individuals while honoring traditional culinary culture besides promoting physical activity, particularly leisure activities. Obesity interventions can be strengthened by focusing on men, the aged, those with lower and higher socioeconomic status, and physical activities and dietary patterns. It would be better to start awareness campaigns in schools and colleges, thus the new generation knows the consequences of being overweight and obese and its preventive strategies, and they can modify their dietary patterns, physical activities, and lifestyle accordingly.

### Limitations

The present study being cross-sectional cannot determine causality. Second, because height and weight are self-reported, they may be underestimated or overstated. Third, because our dietary instrument was designed to offer information about the number of times specific food items were ingested rather than the number of individual food items, we were unable to control the effect of the number of specific food items. Fourth, in the present study, the convenient sampling method was used in which the generalization of the results is compromised. The study's findings can be extrapolated to the population of Saudi Arabia's Makkah region but not the entire country. The likelihood of recall bias in reporting all lifestyle behaviors cannot be ruled out. Fifth, the questionnaire section on food frequency items did not consider portion size, and this factor could have altered the correlations between food habits and being overweight or obese.

## Conclusion

Being overweight and obese were prevalent in 32.8% and 23% of the population, respectively. The current study found few sociodemographic and lifestyle factors related to obesity that could be useful targets for preventing and managing obesity among Saudi inhabitants. Focused intervention strategies are needed to deal with the obesity epidemic. Obesity prevention through active lifestyles and a healthy diet should be a national public health priority. Future research should also focus on implementing a healthy lifestyle and assessing its impact on obesity. Furthermore, government agencies and research organizations must maintain ongoing surveillance of overweight and obesity.

## Data availability statement

The raw data supporting the conclusions of this article will be made available by the authors, without undue reservation.

## Ethics statement

The ethical approval was taken from the Biomedical Ethics Research Committee of King Abdulaziz University, Jeddah, Saudi Arabia (Reference No. 471-21). The patients/participants provided their written informed consent to participate in this study.

## Author contributions

SA contributed to the conceptualization, design, and analysis of the study. MB and TA contributed to the study design, analysis, and preparation of the manuscript. NA, EH, RA, MA, SA, and TA helped in study design, data collection, and interpretation. All authors contributed significantly and approved the submitted version for publication.
